# Evaluation of Pachymetric Measurements with Scheimpflug Photography-Based System and Optical Coherence Tomography Pachymetry at Different Stages of Keratoconus

**DOI:** 10.1155/2014/719205

**Published:** 2014-07-20

**Authors:** Betül İlkay Sezgin Akçay, Engin Bilge Özgürhan, Ercüment Bozkurt, Tuğba Kurt, Yusuf Yıldırım, Mediha Gülen Coşar, Aydın Yıldırım, Jülide Canan Umurhan Akkan, Ahmet Demirok

**Affiliations:** ^1^Umraniye Research and Training Hospital, Eye Clinic, Elmalıkent Mahallesi Adem Yavuz Caddesi No. 1, Umraniye, 34766 Istanbul, Turkey; ^2^Beyoglu Research and Training Hospital, Eye Clinic, Bereketzade Mh., Beyoğlu, 34420 Istanbul, Turkey; ^3^Fatih University, Medical Faculty, Eye Clinic, Orhantepe Mh., Sahil Yolu Sk. No. 16, 34844 Istanbul, Turkey; ^4^Vakif Gureba Research and Training Hospital, Eye Clinic, İskender Paşa Mh., Adnan Menderes Boulevard, 34093 Istanbul, Turkey

## Abstract

The aim of this study was to compare the central and peripheral pachymetric measurements determined with Sirius system and Visante OCT and evaluate the agreement between them at different stages of keratoconus. Measurements were not significantly different in all patients and subgroups and showed high correlation for the corneal thicknesses of the entire cornea in different stages of keratoconus.

## 1. Introduction

Keratoconus is a noninflammatory disorder of the cornea, characterized by the ectasia of the central or paracentral region leading to corneal thinning and surface distortion. The disease is usually bilateral and has its onset at puberty [1]. Progressive corneal steepening, usually at the inferior to the center of the cornea, leads to myopia and irregular astigmatism at early stages and hydrops and corneal scarring in the later stages of the disease. Although the most notable feature of keratoconus is the central and paracentral ectasia, recent studies have revealed that midperipheral portion of the cornea is also effected [2]. In keratoconus, patients use of imaging systems has been important in screening and follow-up. Serial measurements of corneal thickness with topography are important for assessment of progression [3].

Scheimpflug-based, ultrasound (US) based, slit scanning, and optical coherence tomography (OCT) based systems allow the clinician to obtain corneal pachymetric information. Ultrasonic biometry which was the most commonly used method for CT measurements until recently has some limitations. It is a contact procedure; thus it requires corneal anesthesia and aseptic precautions. Also dependence of probe alignment by the operator may diminish reliability of acquiring information about entire cornea [4]. Scheimpflug photography-based, slit scanning topography, and OCT systems are noninvasive procedures for obtaining global corneal pachymetric information [5, 6].

The Visante OCT (Carl Zeiss Meditec, California, USA) which is an optically based method uses low coherence interferometer principle and is designed to evaluate anterior segment structures with a high resolution. The Sirius system (Phoenix, Costruzione Strumenti Oftalmici) is a new topography device which has combination of 2 monochromatic, 360-degree rotating Scheimpflug cameras and a placido disk. Reliable and consistent measurements between devices are crucial because they help clinician in the diagnosis, follow-up, and treatment of keratoconus [7]. The aim of the current study was to compare the central and thinnest point and 3 mm, 5 mm, and 8 mm corneal pachymetric zones in different stages of keratoconus with the Scheimpflug photography-based topography system and the OCT system and also to evaluate agreement between them.

## 2. Subjects and Methods

### 2.1. Subjects

A retrospective chart review was performed on patients with diagnosis of keratoconus. A hundred and sixty-six eyes of 84 patients (47 male, 37 female) with a mean age of 26.38 ± 4.46 were included in the study. Informed consent was obtained from all the patients in accordance with the Declaration of Helsinki before any intervention was performed. Patients with serious corneal scarring, pellucid marginal degeneration, keratoglobus, previous ocular surgery, and any other significant ophthalmic pathology other than keratoconus were excluded from the study.

A complete ophthalmic examination was performed, including visual acuity, manifest refraction, slit lamp biomicroscopy, Goldmann tonometry, and fundus examination. The patients were grouped according to the Amsler-Krumeich keratoconus classification [8] using mean keratometric values from the Sirius topography. The diagnosis of keratoconus had been made based on distortion of the retinoscopic red reflex (scissoring reflex) and keratometric mires, with one of the following biomicroscopic findings: central or paracentral steepening, subepithelial iron accumulation at the base of the cone (Fleischer ring), apical scarring, Vogt's stria, or breaks in the descement's membrane.

### 2.2. Instrumentation

The Sirius system (Phoenix, Costruzione Strumenti Oftalmici) fully analyzes the cornea and anterior segment by using 2 monochromatic, 360-degree rotating Scheimpflug cameras and a small-angle placido disk topographer. Twenty-five Scheimpflug images (meridians) and 1 placido top-view image are needed for a full scan. It provides tangential and axial curvature data of the anterior and posterior corneal surfaces, the global refractive power of the cornea, a biometric estimation of various structures, a corneal wavefront map with an analysis of visual quality, and corneal pachymetry maps. A pachymetric map is constructed by measuring 35 632 points for the anterior and 30 000 for the posterior corneal surface [6].

The Visante OCT (Carl Zeiss Meditec, California, USA) is a time domain OCT system which performs noncontact, high-resolution, cross-sectional, and 3-dimensional imaging of the cornea and the anterior segment of the eye by the analysis of the interference generated between a reference beam and the beam reflected by the different anterior segment structures. It uses a 1310 nm super luminescent diode source for scanning. The system is connected to a computer with specific software which eliminates distortion in the measurements induced by optical factors of transmission, interprets the images, and reconstructs the pachymetry map [6, 9].

### 2.3. Measurements of Corneal Thickness

Measurements were done under the mesopic light condition and repeated with two different optical methods: Visante OCT system (Carl Zeiss Meditec AG) and the Sirius Scheimpflug-based system (Costruzione Strumenti Oftalmici) according to manufacturer's guidelines. Subjects were advised to blink before every scanning in order to provide an optically smooth tear film over the cornea, positioned in the head rest, and they were asked to fixate on the target on the center of the camera without blinking during the scan.

All images and pachymetric maps were analyzed after recording the measurements. The corneal thickness at the geometric center was used as the reference for defining the rest of the locations through the* x-y* Cartesian coordinate grid provided by the software of each instrument. Corneal thicknesses were recorded in all pachymetric maps obtained with each system at the same locations as follows:

1. corneal thickness at the geometric center of the cornea, the central corneal thickness (CCT),
2. the thinnest corneal thickness (TCT),
3. the mean thickness value of 3 mm diameter of the corneal area that surrounds the central corneal thickness (CT 3 mm),
4. the mean thickness value of 5 mm diameter of the corneal area that surrounds the central corneal thickness (CT 5 mm),
5. the mean thickness value of 8 mm diameter of the corneal area that surrounds the central corneal thickness (CT 8 mm).

### 2.4. Statistical Analysis

The Statistical Package for the Social Sciences Statistical Software, version 11.5 (SPSS Inc., Chicago, IL, USA), was used for the statistical analysis. Data was expressed as means ± standard error of the mean. Paired *t*-test was used to analyze the differences between the measurements of the devices and intraclass correlation coefficients (ICCs) were calculated for the 2 repeated measurements. *P* < 0.05 was considered to be statistically significant. The agreement of pachymetric measurements of the Sirius system and the Visante OCT system was evaluated by Bland-Altman analysis. Difference between the measurements with different methods is plotted against their average in a Bland-Altman graphic. The 95% limits of agreement (LoA) (mean difference** ±** 1.96 standard deviation) gave the distance between the measurements by the methods with 95% confidence.

## 3. Results

This study evaluated 166 eyes of 84 patients with a diagnosis of keratoconus. There were 46 men and 38 women with a mean age ± standard deviation of 26.38 ± 4.46 years (range 21–40 years). Two patients have unilateral keratoconus. The mean keratometric value and apex curve (*K*) was 48.13 ± 4.28 Diopter (D) (range 40.73–65.07) and 57.57 ± 11.48 D (range 44.34–153.7), respectively. There were 94 eyes in stage 1 (mean *K* < 48.00 D), 53 eyes in stage 2 (mean *K* < 53.00 D), 6 eyes in stage 3, (mean *K* > 53.00 D), and 13 eyes in stage 4 (mean *K* > 55.00 D) according to the Amsler-Krumeich classification. Patient demographics of whole cohorts and subgroups are given in Table 1.

### 3.1. Comparison of the Corneal Thickness Measurements

Mean CCT, TCT, CT 3 mm, CT 5 mm, and CT 8 mm measurements obtained by the Scheimpflug photography–based topography system and the OCT system were not significantly different in whole cohorts and subgroups (*P* > 0.05). Corneal thicknesses decreased in accordance with the severity of the disease at all measurements. The CCT, TCT, CT 3 mm, CT 5 mm, and CT 8 mm measurements of all the patients and each stage of keratoconus obtained by the two devices, differences between measurements, and *P* values of the comparisons are shown in Table 2.

### 3.2. Correlations between the Pachymetry Methods

**Figure 1:**
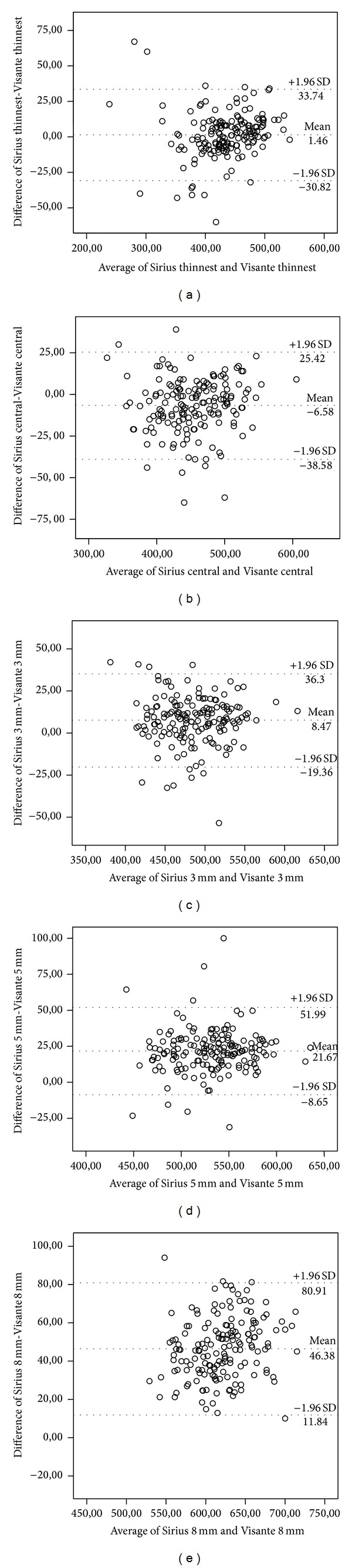
Bland-Altman plot of the differences in TCT (a), CCT (b), CT 3 mm (c), CT 5 mm (d), and CT 8 mm (e) between the Sirius system and the Visante OCT system plotted against the mean value of both. The upper and the lower lines represent the LoA, calculated as mean** ± **1.96 SD.

The correlation coefficients showing the agreements between the CT measurements with Scheimpflug photography-based topography system and the OCT system in the current study are shown in Table 3. The range of difference in the pachymetric measurement between instruments was not constant throughout the cornea. There was a larger range of difference between devices in stages 3 and 4 keratoconus according to stages 1 and 2. When all the patients were considered, the highest correlation coefficient between 2 devices was found to be in the TCT (ICC =  0.946, *P* < 0.01). The highest correlation coefficient was at the TCT (ICC: 0.953, *P* < 0.01) in stage 1, CT 5 mm (ICC: 0.935, *P* < 0.01) in stage 2, CT 5 mm (ICC: 0.977, *P* < 0.01) in stage 3, and CT 5 mm (ICC: 0.900, *P* < 0.01) in stage 4. CT 8 mm measurements showed poorest agreement between devices in all stages (ICC = 0.903 (*P* < 0.01) in stage 1 KC, ICC = 0.866 (*P* < 0.01) in stage 2, ICC = 0.831 (*P* < 0.01) in stage 3, and ICC = 0.731, (*P* < 0.01) in stage 4). Bland-Altman plots of the differences in CCT, TCT, CT 3 mm, CT 5 mm, and CT 8 mm between the Scheimpflug photography-based system and the OCT system plotted against the mean value of both are shown in Figure 1.

## 4. Discussion

OCT imaging systems and corneal topography are used not only to diagnose but also to screen and monitor the progression of the keratoconus [10]. Ultrasonic biometry which was accepted as the main method for evaluating and measuring anterior segment structures has some disadvantages because of being a contact device and high dependence of the operator [11, 12]. Recent corneal topography systems which provide analysis of the thickness throughout the cornea have been practiced widely by recent developments in corneal crosslinking and intracorneal ring segment implantation procedures for keratoconus treatment [13–15]. In the current study we evaluated central and thinnest point and 3, 5, and 8 mm pachymetric measurements obtained with a new corneal topography system which combines Scheimpflug photography and placido disk technologies (Sirius System) and a time-domain OCT system (the Visante system) and also evaluated the agreement of the measurements between two devices in different stages of keratoconus patients.

In our study mean TCT, CCT, CT 3 mm, CT 5 mm, and CT 8 mm measurements in subgroups and whole cohorts are not significantly different between 2 devices (*P* > 0.05). As expected the lowest thickness recorded in all cases was at the central location with both devices. Milla et al. evaluate the intraobserver repeatability of pachymetric measurements obtained with Scheimpflug photography-based system (Sirius system) and optical coherence tomography (Visante OCT) in 18 eyes of 18 patients with no ocular pathology [6]. In contrast to our findings they found significant differences in 2.5 and 4 mm locations of cornea. They also conclude that measurements obtained in Scheimpflug photography-based system were always larger than measurements of OCT system. Ho et al. report similar measurements with the same OCT device and another Scheimpflug photography-based system used in our study and they found lower thickness values with the OCT technique [16]. We detected similar results with the previous studies except in the CCT measurements in which OCT measurements were 6.58 *µ*m higher than Scheimpflug photography-based system considering whole cohorts.

We also evaluated correlation of pachymetric results between two devices. In our study the highest correlation coefficient between two devices was found to be in the TCT. Measurements were less consistent towards the periphery of the cornea with the measurement of the same points in both devices. Similar to our findings Milla et al. reported that measurements 4.0 mm, nasally, were less consistent than central and peripheral 2.5 mm measurements [6]. Dutta et al. compare the central and peripheral pachymetry measurements using optical, ultrasound, and OCT and evaluate agreement between them in 106 eyes of 67 keratoconus patients. Similar to our results they conclude that weak correlations and larger limits of agreement were between techniques in peripheral zones according to central cornea [10]. Huang et al. assessed the repeatability and reproducibility of 3 rotating Scheimpflug cameras and 1 Fourier-domain optical coherence tomography (FD-OCT) system in 66 eyes of 66 volunteers and concluded that precision of CCT, TCT, and CT 2 mm measurements was better than that of CT 5 mm measurements [17]. The cause of repeatability error of peripheral pachymetry may be related to the use of unstable reference point as the center of the pupil instead of vertex cornea and the points captured for the same location decrease from the central to the peripheral cornea with a Scheimpflug photography-based system [18]. In addition to the rotation of the camera around the optical axis the overlap of Scheimpflug images is greater in the center of the cornea according to that of periphery [19]. Many researchers have reported more variability of measurements for peripheral corneal thickness than for CCT [19–21].

In the current study patients were divided into subgroups according to Amsler-Krumeich classification and we confirmed that the CT measurements decrease as the severity of the disease increases. Wolffsohn et al. and Demir et al. also found a negative correlation between stage of keratoconus and corneal thickness [22, 23]. In our study all of the subgroups showed significantly high correlations between measurements of the 2 devices (*P* < 0.001) and the greatest difference was at the CT 8 mm in all stages of keratoconus. Cinar et al. compare (CCT) measurements with a rotating Scheimpflug camera, noncontact specular microscopy, optical low-coherence reflectometry, and ultrasonic pachymetry in different stages of keratoconus patients. Similar to our results they reported that the lowest correlation coefficient was seen at stage 4 keratoconus at each binary measurement of the devices [24].

One of the main limitations of the study is the lack of comparison with US-Pachymetry which is still regarded as gold standard technique in many places. Absence of the intra- and interobserver reproducibility of the measurements obtained with each instrument is another limitation of the current study.

In conclusion, results of this study suggest that despite the higher interdevice variation in peripheral corneal measurements, Sirius system and the Visante OCT system showed high correlation when measuring CCT, TCT, CT 3 mm, CT 5 mm, and CT 8 mm in different stages of the keratoconus. Although CCT measurements have been taken into account for screening of keratoconus in clinical practice, evaluation of the global corneal pachymetric information is crucial because of the higher topographical variations in the presence of ectasia. Further studies with larger sample size are warranted to compare the reliability and reproducibility of the topography devices in keratoconus patients.

## Figures and Tables

**Table 1:** Patient demographics.

	Stage 1 (*n* = 64)	Stage 2 (*n* = 53)	Stage 3 (*n* = 26)	Stage 4 (*n* = 23)	Total (*n* = 166)
Age					
Min/max	21/40	21/39	22/35	21/35	21/40
Mean ± SD	26.35 ± 4.36	26.34 ± 4,58	28,83 ± 4,95	26,23 ± 4,56	26,42 ± 4,45
Sex					
Female (*n*, %)	13 (40.6%)	10 (37.1%)	7 (53.8%)	8 (66.6%)	38 (45.2%)
Male (*n*, %)	19 (59.3%)	17 (62.9%)	6 (46.2%)	4 (33.3%)	46 (54.7%)
*K*1 (D)					
Min/max	38,31/47,25	45,04/51,05	50,01/53,9	52,27/64,05	38,31/64,05
Mean ± SD	43,98 ± 1,67	48 ± 1,57	51,99 ± 1,5	55,95 ± 3,76	46,49 ± 3,99
*K*2 (D)					
Min/max	41,33/63,14	49,38/56,83	55,02/57,51	57,89/68,59	41,33/68,59
Mean ± SD	46,9 ± 2,86	52,04 ± 1,58	56,84 ± 0,94	61,09 ± 3,58	50,01 ± 4,95
Mean keratometric value (D)					
Min/max	40,73/47,99	48,07/52,85	53,23/54,94	55,04/65,07	40,73/65,07
Mean ± SD	45,31 ± 1,69	49,93 ± 1,37	54,28 ± 0,64	58,39 ± 3,47	48,13 ± 4,28
*K* apex (D)					
Min/max	44,34/85,9	54,02/67,24	60,7/74,18	63,08/153,7	44,34/153,7
Mean ± SD	53,12 ± 6,03	59,06 ± 3,29	64,75 ± 4,82	80,35 ± 26,86	57,57 ± 11,48

**Table 2:** Comparative analysis of the Sirius system and the Visante OCT measurements in different stages of keratoconus.

Measurement localizations	Sirius system	Visante OCT	Difference between 2 measurements	*P*
	Mean ± SD	Mean ± SD	Mean ± SD	
Stage 1				
TCT (*µ*m)	457,63 ± 45,03	454,83 ± 41,98	2,8 ± 13,36	**0,675**
CCT (*µ*m)	473,79 ± 43,46	478,37 ± 41,02	−4,59 ± 14,2	**0,202**
CT 3 mm (*µ*m)	502,60 ± 40,86	495,62 ± 38,26	6,98 ± 13,81	**0,121**
CT 5 mm (*µ*m)	546,77 ± 40,52	526,56 ± 37,86	20,21 ± 16,62	**0,186**
CT 8 mm (*µ*m)	643,72 ± 46,99	598,03 ± 40,02	45,69 ± 19,21	**0,225**
Stage 2				
TCT (*µ*m)	418,04 ± 35,72	418,74 ± 36,96	−0,7 ± 18,83	**0,788**
CCT (*µ*m)	431,26 ± 34,93	441,13 ± 38,37	−9,87 ± 18,27	**0,685**
CT 3 mm (*µ*m)	477,85 ± 31,45	470,35 ± 34,96	7,5 ± 14,09	**0,754**
CT 5 mm (*µ*m)	535,36 ± 33,45	514,83 ± 35,52	20,53 ± 12,44	**0,465**
CT 8 mm (*µ*m)	641,05 ± 37,25	594,84 ± 35,96	46,21 ± 15,2	**0,157**
Stage 3				
TCT (*µ*m)	410,17 ± 28,27	404,17 ± 26,56	6 ± 10,88	**0,235**
CCT (*µ*m)	423,50 ± 29,27	421,33 ± 28,66	2,17 ± 11,96	**0,676**
CT 3 mm (*µ*m)	485,75 ± 28,81	463,05 ± 26,41	22,71 ± 5,52	**0,248**
CT 5 mm (*µ*m)	555,47 ± 26,52	521,27 ± 24,05	34,2 ± 5,39	**0,354**
CT 8 mm (*µ*m)	652,49 ± 30,18	601,69 ± 17,11	50,8 ± 14,27	**0,421**
Stage 4				
TCT (*µ*m)	353,23 ± 34,77	354,69 ± 40,24	−1,46 ± 26,56	**0,846**
CCT (*µ*m)	380,54 ± 23,34	392,15 ± 33,91	−11,62 ± 21,36	**0,774**
CT 3 mm (*µ*m)	453,62 ± 23,29	436,92 ± 23,15	16,7 ± 15,03	**0,458**
CT 5 mm (*µ*m)	534,60 ± 30,90	503,46 ± 28,48	31,13 ± 13,28	**0,455**
CT 8 mm (*µ*m)	648,28 ± 36,15	598,22 ± 33,88	50,05 ± 17,11	**0,154**
Total				
TCT (*µ*m)	435,10 ± 50,90	433,63 ± 49,12	1,46 ± 16,47	**0,254**
CCT (*µ*m)	451,09 ± 48,52	457,67 ± 47,32	−6,58 ± 16,34	**0,847**
CT 3 mm (*µ*m)	490,25 ± 39,49	481,77 ± 39,91	8,48 ± 14,2	**0,735**
CT 5 mm (*µ*m)	542,49 ± 37,50	520,81 ± 36,55	21,67 ± 15,25	**0,622**
CT 8 mm (*µ*m)	643,54 ± 42,55	597,16 ± 37,49	46,38 ± 17,62	**0,128**

*P*: paired samples *t*-test, SD: standard deviation.

CCT: central corneal thickness, TCT: thinnest corneal thickness, CT 3 mm: corneal thickness at 3 mm, CT 5 mm: corneal thickness at 5 mm, and CT 8 mm: corneal thickness at 8 mm.

**Table 3:** Degree of correlation between measurements of two devices at different stages of the keratoconus.

Measurement localizations	Stage 1		Stage 2		Stage 3		Stage 4		Total	
	ICC (*r*)	%95 CI	ICC (*r*)	%95 CI	ICC (*r*)	%95 CI	ICC (*r*)	%95 CI	ICC (*r*)	%95 CI
TCT	0,953∗∗	0,930–0,968	0,914∗∗	0,855–0,949	0,921∗∗	0,547–0,989	0,751∗∗	0,363–0,917	0,946∗∗	0,927–0,960
CCT	0,944∗∗	0,916–0,962	0,876∗∗	0,794–0,926	0,915∗∗	0,518–0,988	0,881∗∗	0,656–0,962	0,942∗∗	0,922–0,957
CT 3 mm	0,939∗∗	0,910–0,959	0,910∗∗	0,849–0,947	0,866∗∗	0,866–0,997	0,791∗∗	0,446–0,931	0,936∗∗	0,914–0,953
CT 5 mm	0,910∗∗	0,868–0,939	0,935∗∗	0,890–0,962	0,977∗∗	0,849–0,997	0,900∗∗	0,706–0,968	0,915∗∗	0,887–0,937
CT 8 mm	0,903∗∗	0,858–0,935	0,866∗∗	0,778–0,920	0,831∗∗	0,204–0,974	0,731∗∗	0,325–0,909	0,903∗∗	0,871–0,928

ICC: intraclass correlation coefficient, CI: confidence interval, **P < 0.01 CCT: central corneal thickness, TCT: thinnest corneal thickness, CT 3 mm: corneal thickness at 3 mm, CT 5 mm: corneal thickness at 5 mm, and CT 8 mm: corneal thickness at 8 mm.
